# One in six physiotherapy practices in primary care offer musculoskeletal ultrasound – an explorative survey

**DOI:** 10.1186/s12913-020-05119-3

**Published:** 2020-03-24

**Authors:** Margit K. Kooijman, Ilse C. S. Swinkels, Bart W. Koes, Dinny de Bakker, Cindy Veenhof

**Affiliations:** 1 Department of Allied Health Care, NIVEL Netherlands Institute of Health Services Research, PO BOX 1568, 3500 BN Utrecht, the Netherlands; 2 Department of General Practice, Erasmus Medical Center, Rotterdam, the Netherlands; 3 Scientific Centre for Transformations in Care and Welfare (TRANZO), Tilburg University, Tilburg, the Netherlands; 4 Department of Rehabilitation, Nursing Sciences and Sport, University Medical Center Utrecht, Utrecht, the Netherlands

**Keywords:** Musculoskeletal pain, Shoulder pain, Ultrasonography, Physical therapy modalities

## Abstract

**Background:**

The first aim of this research was to investigate the current prevalence of musculoskeletal ultrasound in Dutch physiotherapy practices. The second aim was to explore experiences of physiotherapists with musculoskeletal ultrasound in a primary care setting with patients presenting with shoulder complaints.

**Methods:**

A random sample of 1000 owners of primary care physiotherapy practices was sent a questionnaire to investigate the prevalence of musculoskeletal ultrasound. A second questionnaire was sent to physiotherapists using musculoskeletal ultrasound to explore experiences with it in patients with shoulder complaints.

**Results:**

The net response rate of the first questionnaire was 57.7%. In 18% of the physiotherapy practices musculoskeletal ultrasound was offered. Sixty-nine physiotherapists returned the second questionnaire. Physiotherapists indicated they most often used musculoskeletal ultrasound in patients with shoulder complaints, mainly for suspected tissue damage (83.7%), followed by making a diagnosis (63.3%) and for determining the choice of treatment (36.7%). Physiotherapists reported the biggest advantage was that they were better able to diagnose presenting shoulder complaints. The most frequently mentioned disadvantage of the use of musculoskeletal ultrasound was that assessment is difficult and that there is a risk that findings may not be sufficiently linked to history and physical examination.

**Conclusion:**

One in six physiotherapy practices in the Netherlands offer musculoskeletal ultrasound. It is mainly used for patients with shoulder complaints, with an emphasis on detecting tissue damage and as an aid for diagnosis. Physiotherapists trained to work with musculoskeletal ultrasound seem enthusiastic and are at the same time aware of its disadvantages.

## Background

Musculoskeletal ultrasound (MSU) in secondary care has become a patient friendly, accurate and cost-effective method for diagnosing shoulder complaints [1]. In recent years, it also gained popularity amongst GPs and physiotherapists. However, basic data on MSU in primary care is scarce. For example, the uptake, the targeted patient population and reimbursement is largely unknown [2–4]. For patients, the possibility of accurate additional imagery in combination with physical examination at one place close to their homes is attractive. For policy makers, it is of importance in the discussion of substitution from secondary to primary care. Scholten-Peeters (2014) investigated the opinions and experiences of Dutch radiologists and orthopedic surgeons about the use of MSU in primary care [3]. It shows that they had little confidence in its use in primary care and believed that diagnostic MSU belongs in secondary care. The discussion on the desirability of MSU in primary care, calls for a further investigation on actual prevalence and experiences of MSU physiotherapists.

It is suspected by the authors that for a large part, MSU is used to diagnose shoulder complaints. Therefore, this paper focuses mainly on patients with shoulder problems. Except for back and neck problems, shoulder pain is the most frequent complaint in physiotherapy practice [5]. Despite its frequent occurrence, studies report unfavourable outcome in many patients. In physiotherapy practice, the percentage of patients recovering after treatment varies from 20 to 79% and it is known that the treatment duration is relatively long [6]. This is frustrating for patients and clinicians and leads to high costs both for secondary care and sick leave [7–9].

Since long-lasting complaints contribute to an unfavourable prognosis, an adequate and quick diagnosis is important [10, 11]. This is the starting point for choosing the right treatment with the appropriate clinician, most frequently the GP or physiotherapist. However, the shoulder is one of the most complex joints to diagnose complaints correctly. In clinical practice and in research, history and physical tests are relied on for this purpose. Yet, many studies show that these physical tests, even when combined, have limited diagnostic value [12]. In search for an alternative, MSU as a diagnostic aid is on the rise in primary care [13]. The important question being how physical tests and MSU relate to one another and whether MSU is considered valuable by clinicians in improving the management of shoulder conditions. As a starting point, the current study explores the use of MSU in the clinical practice of the physical therapist. As such, it addresses two research questions. Firstly, what is the current prevalence of MSU in Dutch physiotherapy practices? Secondly, what are the experiences of MSU physiotherapists with MSU in a primary care setting in patients with shoulder complaints?

## Methods

We performed an explorative survey of a sample of owners of physiotherapy practices and MSU physiotherapists in the Netherlands.

### Design and participants

#### Practice owners

A random sample of 1000 owners of physiotherapy practices in the Netherlands, representative of age, gender, type of practice and degree of urbanicity were invited to participate to investigate the diffusion of MSU in physiotherapy practices by means of a questionnaire. They were recruited from the national register database for physical therapists of the Dutch Institute for Health Services Research (NIVEL). At the end of the questionnaire, they were asked to state the names and email addresses of MSU physiotherapists working in their practice, if any.

#### MSU physiotherapists

In a second questionnaire, these MSU physiotherapists were questioned on their opinion and experience with MSU. Since MSU is relatively new in physiotherapy settings, a random sample to recruit more participants did not seem appropriate. It was therefore decided to recruit additional MSU physiotherapists through snowball-sampling in the authors professional network and through requests on social media. According to the Dutch Medical Research Involving Human Subjects Act, this study did not require ethics approval. The study did follow the Declaration of Helsinki research ethics [14].

### Data collection

#### Practice owners

The questionnaire for practice owners included questions on the characteristics of the practice and on reasons for offering or non-offering MSU. A group of researchers, pioneers in the field of MSU education and from research institute NIVEL, and MSU physiotherapists (*n* = 12) was asked to provide feedback on the scope and completeness of the questionnaire. Modifications were made and the final version was tested in another group (*n* = 4) to evaluate feasibility. The final digital questionnaire consisted of 18 open and close-ended questions and took approximately 7 min to complete. A letter with online login to the questionnaire was sent to all participants by letter post. After 2 weeks, all non-responders received the questionnaire by post. After 4 weeks, all non-responders received a reminder by post.

#### MSU physiotherapists

The digital questionnaire for MSU physiotherapists comprised of four sections: general information on the respondents, opinions and experiences with MSU in physiotherapy practice for the general patient population and for patients with shoulder complaints specifically, advantages and disadvantages and several propositions. It consisted of 33 open and close-ended questions and 13 propositions and took approximately 10 min to complete. The same check on scope, completeness and feasibility was carried out before it was sent to all respondents by email. Reminders (by email) were sent after one and 3 weeks.

### Data analysis

Open-ended questions were read first, then summarized by topic by the first author. This grouping was discussed with two co-authors until consensus was reached. As for the practice owner questionnaire, non-response analyses were performed using t-tests and Chi-square tests (a = 0.05). To test differences between practices with and without MSU, Chi-square tests were used for categorical data and two-sample t-tests were used for continuous data. Data was checked for normal distribution.

## Results

### Practice owners

#### Respondents

Of the 1000 questionnaires distributed, 30 were returned because of incorrect addressing. In total, 560 responders completed the questionnaire of which 197 digitally and 363 by letter post. The net response rate was 58%. Table 1 presents the characteristics of responding and non-responding practice owners. It shows that respondents were statistically older in age than non-respondents, otherwise groups were comparable.

**Table 1:** Characteristics of invited physiotherapy practice owners and results of non-response analysis

	Respondents (*n* = 560) (%)	Non-respondents (*n* = 410) (%)	*P* value non-response analysis
Gender (% male)	65.8	64.4	0.645
Age (mean, sd)	54.9 (7.8)	53.2 (8.7)	0.001
Type of practice:			0.182
Solo	32.6	37.1	
Duo	13.8	15.3	
Group	53.7	47.7	
Degree of urbanicity:			0.705
Urban	46.0	46.5	
Suburban	21.5	19.4	
Rural	32.6	34.1	
Region:			0.245
North	8.8	12.4	
East	19.5	18.6	
West	45.6	46.3	
South	26.1	22.8	

MSU was offered in 18% (*n* = 99) of the practices. These practices were bigger in number of full-time equivalent (FTE) and in number of physiotherapists with a specialty in pelvic, manual, sports or occupational physiotherapy than in practices not offering MSU (Table 2). On average, there were 2.0 (SD 1.0) MSU physiotherapists working in a practice offering MSU.

**Table 2:** Characteristics of participating practices

Variable	Total	Practice with MSU (*n* = 99) (%)	Practice without MSU (*n* = 461) (%)	*P* value
Number of fte (mean, sd)	3.6 (3.4)	6.0 (4.0)	3.1 (3.0)	< 0.001
Specialty:				
Pelvic	19.8	31.3	17.2	0.001
Geriatrics	8.9	13.1	8.0	0.100
Pediatrics	21.4	28.3	19.9	0.064
Manual	61.1	85.9	55.6	< 0.001
Orofascial	7.1	11.1	6.2	0.083
Psychosomatic	15.9	20.2	15.0	0.201
Sports	26.6	53.5	20.8	< 0.001
Edema	31.0	40.4	28.9	0.025
Occupational	10.3	25.3	7.1	< 0.001
MSU PT				< 0.001
yes	18.5	92.8	2.5	
no	81.5	7.2	97.5	

#### Reasons for (non) offering MSU

Of the practice owners who offer MSU, 92% indicated that they would purchase MSU equipment when given the choice again. On the open-ended question ‘what is/are the main reason(s) for purchasing MSU equipment’, most answers could be attributed to the improvement of diagnosis. High costs for purchase/ no reimbursement and not using it at all were mentioned by those who would not choose for MSU again. Of the practices without MSU, 34% did not have a specific reason, 7% did not have MSU equipment yet but thought of purchasing it and 59% had specific reasons for not offering MSU. These included high costs and no reimbursement; not suitable for the practice’ patient population; doubts on the scientific evidence or benefit for daily practice; MSU does not fit in the professional profile of the physiotherapist; no need because of co-operation with another MSU practice or resistance of GPs (open-ended question: ‘Is/are there specific reason(s) for not offering MSU in your practice (yet)?’).

### MSU physiotherapists

#### Respondents

In total, 69 MSU physiotherapists reacted on our request to fill out a questionnaire on the use of MSU. Table 3 presents the characteristics of participating physiotherapists.

**Table 3:** Characteristics of MSU physiotherapists (*n* = 69)

	(%)
Gender (male)	84.1
Age, mean (sd)	45.3 (11.4)
Experience as physiotherapist, mean years (sd)	21.7 (11.0)
Specialty:	
Pelvic	1.5
Geriatrics	0.0
Pediatrics	0.0
Manual	50.0
Orofascial	1.5
Psychosomatic	0.0
Sports	16.2
Edema	4.4
Occupational	7.4
Year MSU education completed:	
< = 2006	20.0
2007–2010	33.9
> =2011	46.1
Masterclass on shoulder disorders (yes)	53.5
Experience with MSU, mean years (sd)	4.4 (3.2)

On the question how reimbursement was arranged, 63% of the respondents indicated MSU was claimed as a regular physiotherapy treatment, 37% did not claim (additional) costs at all because they considered it part of treatment and nine respondents did not answer the question. Almost all MSU physiotherapists agreed that treatment has become more efficient because of MSU and 76% thinks it has reduced costs. On the open-ended question: ‘for which part of the body do you use MSU most frequently?’ 71% of the MSU physiotherapists indicated they focused on patients with shoulder problems, another 20% focused on shoulder and lower extremity. Almost 62% of the MSU physiotherapists thought that patients specifically chose to visit their practice because of the possibility of MSU treatment and 80% agreed with the proposition that patients were more satisfied because of it.

#### Opinion and experience with MSU

Of the respondents, 89% (*n* = 58) used MSU in daily practice. These respondents were asked several questions on their use of MSU in physiotherapy practice for the general patient population and for patients with shoulder complaints specifically.

In 37% of the new patients with shoulder complaints and in 4% of new patients in general, MSU is often (in > 75% of the patients) or always used (Table 4). Almost 77% of the MSU physiotherapists agree with the proposition that ideally MSU should be used in all patients with shoulder complaints.

**Table 4:** Opinions and experiences of MSU physiotherapists (%) (*n* = 58)

	Never^a^	Sometimes	Regularly	Usually	Often	Always
How many times do you perform an echo in new patients with shoulder complaints?	4.1	8.2	20.4	30.6	26.5	10.2
How many times do you perform an echo in the general patient population?	0.0	44.9	40.8	10.2	4.1	0.0
How often does your initial diagnosis change in patients with shoulder complaints?	4.3	48.9	38.3	4.3	4.3	0.0
How often does your initial diagnosis change in the general patient population?	0.0	56.3	43.7	0.0	0.0	0.0
How often do you receive requests for MSU from colleagues for patients with shoulder complaints?	4.2	20.8	52.1	14.6	8.3	0.0
How often do you receive requests for MSU from colleagues for the general patient population?	2.1	41.7	43.7	4.2	8.3	0.0

^a^never: in 0% of patients, sometimes: 1–25%, regularly: 26–50%, usually: 51–75%, often: 76–99%, always: 100%

In patients with shoulder complaints, MSU is mainly used for suspected tissue damage (84%) (Table 5). About half of the respondents indicated that the results of the MSU scan regularly changed their initial diagnosis among patients with shoulder complaints (Table 4). These results are much the same in the general patient population. Over 90% of the MSU physiotherapists indicated that they feel more confident in their choice of treatment because of MSU and 65% considered their treatment improved because of it.

**Table 5:** Main purposes of MSU

	Patients with shoulder complaints (%)	General patient population (%)
I use MSU mainly for:		
Reassurance of the patient	16.3	24.5
Choice of treatment	36.7	36.7
Adjustment of treatment	8.2	12.2
Evaluation of treatment	28.6	24.5
Doubts of diagnosis	28.6	18.4
Making a diagnosis	63.3	75.5
Suspicion on tissue damage	83.7	75.5
Indication for physiotherapy	12.2	12.2

More than 50% of the respondents receive regular requests for MSU from colleagues in patients with shoulder complaints (Table 4). Another 23% receive these requests often to always. In the general patient population around 44% of the MSU physiotherapists never or sometimes receive these requests. For both populations, it mainly concerns requests from general practitioners and colleagues from their own physiotherapy practice and in both populations 25% use history and clinical information provided by the applicant without examining the patient themselves.

#### Advantages and disadvantages

Regarding the open-ended question as to the biggest advantage of MSU, physiotherapists most frequently indicated its role in better diagnosing shoulder complaints, which helps them with prognosis and treatment. Almost 90% agreed with the proposition that dynamic examination is the most important advantage compared to other diagnostic imaging. The most frequently mentioned disadvantage was that assessment is difficult and that there is a risk that findings may not be sufficiently linked to history and physical examination. Because physiotherapists’ central starting point is the patient with his complaints, many MSU physiotherapists (85%) first performed history and physical examination and used MSU additionally. However, more than 65% disagreed that history and physical examination are more important than MSU findings. When clinical findings contradict results of MSU, 21% trusted MSU, 32% discussed it with a colleague or GP, 14% trusted the clinical findings, 6% directly referred to the GP and 27% indicated their strategy depended on the particular findings. Of the MSU physiotherapists, 66% advised patients to contact their GP more quickly and 95% indicated they directed patients to the GP more specifically.

## Discussion

The purpose of current study was to investigate the current prevalence of MSU in Dutch physiotherapy practices and to explore experiences of MSU physiotherapists with MSU in a primary care setting in patients with shoulder complaints. It shows that in 18% of the practices MSU was offered, mainly with the intention to improve diagnosis. Most practice owners seem content with their decision since nine out of ten would make the choice for MSU again. Data on this topic is scarce but research in Australia shows that requests by GPs for diagnostic shoulder ultrasonography are on the rise [15, 16].

The participating MSU physiotherapists in the second part of the study indicate that by far, MSU is most frequently used in patients with shoulder complaints. The most frequently mentioned perceived advantage is that MSU helps them to make a better diagnosis. In new patients with shoulder complaints, MSU is more often used than in general patient population and ideally, many responding MSU physiotherapists think it should be used in all new shoulder patients. This finding supports our observation that in this specific group of patients, responding physiotherapists often seek assurance to improve their diagnosis and/or treatment by using MSU. Apparently, this applies to other clinicians as well since MSU physiotherapists receive relatively many requests from colleagues and GPs for these patients. A quarter of MSU physiotherapists did not examine these referred patients themselves by means of history and physical examination. It is not known to us what pre-existing information the responding MSU physiotherapists would have possessed among their patients, although research amongst Australian GPs indicates that around a third of the MSU requests did not contain any additional information for the radiologist [17]. Since supposed pathology in MSU findings may be asymptomatic, especially in patients over 60 years old, this lack of information could undermine the security that is looked for [18]. Even more so because it has been suggested that MSU is most effective when linked to history and clinical examination by the same clinician [15]. Radiologists do not examine patients themselves but MSU physiotherapists can, hence the profession could actually change this ‘problem’ to their advantage.

Responding MSU physiotherapists themselves stated that dealing with inconsistent findings from MSU and physical examination is difficult. It is the biggest disadvantage from their point of view. This also shows in the diverse strategies they indicate they practice when it happens; some trust the results from MSU, others rely on clinical examination or discuss it with a colleague or GP. This might also explain why MSU physiotherapists more often and more specifically refer patients back to their GP. Whether this eventually leads to an increase or decrease in requests for care and associated costs is a legitimate question for further research.

It is known that MSU is a valid and reliable method to identify full- and partial thickness tears of the tendon if performed by radiologists and/or orthopaedic surgeons, there is only limited evidence for tendinopathy, calcification and bursitis [1, 19, 20]. The first, small sample reliability study amongst physiotherapists in primary care indicates that there is slight to moderate agreement between MSU physiotherapists and radiologists and moderate to substantial agreement between MSU physiotherapists mutually, although both vary depending on pathology and experience [21]. It was also concluded that this was relatively low compared to reliability between radiologists. In their study on opinions on use of MSU in primary care, Scholten-Peeters et al. (2013) found that participating radiologists and orthopaedic surgeons found more disadvantages than advantages including false negative and positive results, lack of experience and not able to relate MSU to other additional imaging and insufficient education [3]. It has been shown that clinicians other than radiologists such as rheumatologists and orthopaedic surgeons are able to achieve comparable levels of diagnostic accuracy [22]. However, additional studies are required to confirm or refute these arguments.

As with direct access physiotherapy, which was another shift in health services and possible substitution from GP care to physiotherapy care and also feared and criticised mainly by other health care professionals, reservations should be taken seriously [23]. The uptake of direct access was on the rise even before it was arranged officially and before (pilot) research was conducted on possible successes and failures. The utility of direct-access physiotherapy was supported by the high percentage of patients accessing this form of healthcare provision [23]. It appears that the profession anticipated and responded well on this changed demand. A similar situation now arises with MSU by physiotherapists; the uptake is on the rise, other health care professionals are sceptical and research is scarce [3]. At the same time, responding MSU physiotherapists appear enthusiastic at offering MSU; they think patients choose their practice specifically and are more satisfied. In addition, they think their treatment is more efficient and they are better able to cure patients. With direct access, new policy was made on education, reimbursement and interdisciplinary communication. The same is desired and required for MSU, also because of the large group of patients that comes via direct access. Objections and difficulties such as conflicting findings should be appointed so that they can be discussed and addressed as important training issues. Furthermore, more research is necessary. First on reliability, which would include intra- and interrater agreement between MSU physiotherapists mutually and between MSU physiotherapists and radiologists. Second, the effectiveness of additional MSU compared to the current situation should be investigated. This includes the desirability of MSU by the profession itself since a substantial group of practice owners indicated that they do not offer MSU, some for a very specific reason such as high costs whilst others mentioned no reason as to why they did not offer it. All in all, the professional need for an alternative for diagnosing patients with shoulder complaints and the possibilities that MSU offer for physiotherapists and their patients and eventually policy makers, should be explored more fully.

### Study limitations

One of the purposes of current study was to explore the experiences of MSU physiotherapists with MSU within Dutch primary care settings in patients with shoulder complaints. Because little is known on MSU in primary care and in a physiotherapy setting particularly, questions were asked about the use of MSU in the general patient population (non shoulder). This was done not with the intention to compare both groups but to outline a framework to better understand the role of MSU in patients with shoulder complaints. However, since results show that MSU is used mainly for shoulder complaints, the differences found between both populations might exist but may be of slight importance in daily practice and are probably based on a small number of patients.

A second limitation of current study is that we measured stated rather than actual practice. Meaning responding MSU physiotherapists might have given socially acceptable answers, for example on delicate matters such as reimbursement. However, a substantial group indicated not doing their own physical examination when another clinician requests MSU, which is not in line with protocol. It also means that they were required to give estimates, for example on the number of patients they see, use MSU or in which they switch diagnosis. Despite this subjectivity, the results indicate an overall trend towards a positive opinion on the use of MSU. At the same time they show that MSU physiotherapists are aware of disadvantages such as the issue of what to do with conflicting results.

## Conclusion

The results from our questionnaires show that 18% of the physiotherapy practices use MSU, mainly for patients with shoulder complaints and with an emphasis on detecting tissue damage and as an aid for diagnosis. MSU physiotherapists seem enthusiastic and are at the same time aware of its disadvantages.

## Data Availability

The dataset supporting the conclusions of this article is included within the article. A copy of the questionnaire can be obtained from the first author.

## Abbreviations

GP: General practitioner
MSU: Musculoskeletal ultrasound
